# Dr. George W. Cook, D.D.S.

**Published:** 1914-02-15

**Authors:** 


Dr. George W. Cook, D.D.S.:—Dr. Cook passed away at his home in Hebron, Indiana, December 21st, 1913. He was stricken with apoplexy at one o'clock in the morning and died about nine o'clock the same evening. Dr. Cook was born in Kentucky in 1865, but his parents moved to Harrisburg, Ills., when he was but two years of age. His father and mother both died before he was eight years old, and George went to live with a physician, Dr. Hastings of Carbondale, Ills., where he acquired a taste for the study of medicine, which however he never was able to gratify. He associated himself with the Southern Illinois Insane Asylum when he was only sixteen years of age and made much of his opportunity to study drugs and their application to the treatment of diseases. He attended the Northwestern Dental School of Chicago one year and then transferred to the Dental Department of Iowa University, where he graduated in 1890. He returned to Chicago and opened an office in Chicago on 47th Street where he continued in practice up to the time of his death. He identified himself with his local, state and the national societies and contributed many papers and much to the discussions of all these organizations. Shortly after beginning practice he took up the study of bacteriology and pathology and made himself a recognized authority on these subjects in their dental application. He was for a number years professor of Dental Bacteriology and Pathology in the Dental School of the University of Illinois. He was Dean of the Dental School for five or six years. He was not only a devoted student but he had a keen appreciation of humor and he made himself universally beloved because of his gracious friendliness. He was a great admirer of men in the professions who were doing scientific work that could be applied to dentistry and his greatest ambition was to become a scientific research worker for his profession. He spent much of his time in doing research work without adequate facilities or the institutional support which is necessary to successfully carry on this kind of work.

Our literature of the past fifteen years contains many of his papers and society discussions. He was very popular with his professional friends in Chicago, and it seems that everybody must know George Cook. He was a commanding figure in every gathering of dentists when he was present. His jovial good nature made him the friend of every one, and it won't seem like the same place to visit Chicago again and not be greeted by his beaming countenance and sincere hand grip.

Dr. Cook was a devoted member of Delta Sigma Delta Fraternity and was a prominent factor in its work and deliberations.

He was for several years the editor of the *American Journal of Dental Science*, and did much valuable work on this journal as editor and contributor.

It is strange to see how a man with so meager a chance, can by pluck and desire do so much in a brief life. All that Dr. Cook has done he has acquired first by indomitable energy and hard painstaking effort. A spirit like his with adequate support and competent advice could have accomplished much more than he has for a young profession like dentistry, and it is just such men as he who by sincere devotion and willing sacrifice of personal ends have helped to make a science of dentistry, when the natural tendency is so strong towards an art craft. We shall miss our friend in the public places of the future, but we are glad that he came into our personal acquaintance as he has made our life more happy and he has inspired us to some higher ideals. We can't have too many such men and his death is a great loss both socially and professionally.

In the autum of 1907, Dr. Cook married Miss Margaret McGill of Hebron, Indiana, and made that place his home, keeping his practice in Chicago, on 47th Street.

